# The Use of Social Media by Clinical Nurse Specialists at a Tertiary Hospital: Mixed Methods Study

**DOI:** 10.2196/45150

**Published:** 2023-08-24

**Authors:** Alya AL-Rumhi, Samira AL-Rasbi, Aaliyah M Momani

**Affiliations:** 1 Nursing Department Clinical Nurse Specialist Sultan Qaboos University Hospital A`Seeb Oman; 2 Child and Maternal Health Department Faculty of Nursing Applied Science Private University Amman Jordan

**Keywords:** social media, clinical nurse specialist, cross-sectional, tertiary hospital, Oman, health education tool

## Abstract

**Background:**

Recently, many health care professionals, who use social media to communicate with patients and colleagues, share information about medical research and promote public health campaigns.

**Objective:**

This study aimed to examine the motives, barriers, and implementation of social media use among clinical nurse specialists in Oman.

**Methods:**

A mixed methods study was conducted among 47 clinical nurse specialists at Sultan Qaboos University Hospital between November and December 2020. Qualitative data were collected using an open-ended questionnaire and analyzed using thematic analysis, and quantitative data were collected with a questionnaire and analyzed using SPSS (version 21.0; IBM Corp).

**Results:**

Of the 47 clinical nurse specialists surveyed, 43 (91.5%) responded. All respondents reported using social media applications, with WhatsApp being the most commonly used platform. Most respondents (n=18, 41.9%) spent 1-2 hours per day on social media. The main motives for using social media were increasing knowledge, communication, reaching patients easily, and reducing the number of hospital visits. The main barriers to social media use were privacy concerns, time constraints, and a lack of awareness of legal guidelines for social media use in the workplace. All participants requested clear rules and regulations regarding the use of social media among health care providers in the future.

**Conclusions:**

Social media has the option to be a powerful institutional communication and health education tool for clinical nurse specialists in Oman. However, several obstacles must be addressed, including privacy concerns and the need for clear guidelines on social media use in the workplace. Our findings suggest that health care institutions and clinical nurse specialists must work together to overcome these impediments and leverage the benefits of social media for health care.Bottom of Form

## Introduction

Several previous studies have revealed that the rapid development of technology has influenced every aspect of human lives, including health care services. The presence of the latest technologies among health care professionals (HCPs) has improved the services provided to their patients [1]. Social media (SM) has become a popular tool for HCPs to manage and interact with patients, promote healthy behaviors, and develop professional network [2-4]. In addition, the use of SM platforms by HCPs is increasing globally [5,6]. Studies have shown that SM can effectively improve health care outcomes and debate issues in health care policy and practice [7]. Furthermore, SM can be used by HCPs to educate and motivate patients, track progress, answer patient queries, and increase patient service satisfaction [8-11]. Scholars have noted that SM tools can be effective in meeting individual and population health needs, especially during the COVID-19 pandemic [8,12]. However, there are very few studies on reflections of SM use among HCPs.

A survey of 658 Chinese nurses found that all participants were SM users, with 84.5% of them believing that SM positively influenced their clinical practice [13]. Furthermore, Wang et al [13] found that nurses use SM for a variety of purposes ranging from receiving messages from work and news to relaxing and networking. SM’s applications in health care have been used to engage with the community through providing mutual education and interaction with patients, caregivers, students, and colleagues [14,15]. Studies have shown that patients want to communicate with their doctors or clinic through SM for sending information regarding laboratory investigations, reports, scheduling appointments, and reminders and for receiving responses to their queries [16]. Furthermore, SM has also been shown to have an impact on rapid information and knowledge dissemination to the people who need it most during the COVID-19 pandemic [8,17].

However, the expansion of SM platforms carries potential risks to HCPs and patients in regard to the dissemination of misleading information, risks to patient confidentiality, the violation of personal-professional limitations, and the potential for the impairment of the professional image [17-19]. There are also potential legal issues, such as maintaining privacy and the lack of control and oversight that could have a negative impact on patient satisfaction [20].

In the Middle East, there is potential for SM to be adopted to improve patient outcomes [21]. A survey by the Centre for Statistics and Information in Oman revealed that the average daily use of SM among Omanis was 6 hours a day. WhatsApp was the most commonly used platform among students, while Snapchat and Facebook were the least used [22]. The Arab Social Media Forum highlighted that the high demand for SM in Oman comes with huge amounts of potential, particularly because SM users account for 77% of the total population, and user growth rates continue to rise [23]. According to Hagg et al [24] and Rahmat et al [25], there is a need for research on SM to expand the geographical focus and test strategies to facilitate the effective and efficient use of social media for health care purposes in the Middle East.

There are 47 clinical nurse specialists (CNSs)—a category of advanced practitioner nurse (APN)—at Sultan Qaboos University Hospital (SQUH). APNs are considered the first line of HCPs when it comes to communicating with patients [26]. APNs play a crucial role in patient care, and their ability to communicate with patients and provide timely and accurate information is essential in improving patient outcomes. According to the National Association of Clinical Nurse Specialists, CNSs are integral to the delivery of cost-effective, high-quality, and patient-centered care [27-29].

However, how SM should be used among CNSs in Oman is not yet well defined. Most of the literature investigated SM usage in nursing or medical education. In addition, there is a dearth of knowledge to understand nurses in general and CNSs in particular and their use of SM in the Middle East and Oman. Therefore, in response to this gap in knowledge, the aim of this study was to examine the use of SM applications among CNSs at SQUH and the motives and barriers that CNSs are facing in implementing SM usage.

## Methods

### Study Design and Setting

A mixed methods design was used to collect data to uncover the best practices of CNSs in terms of SM use. A paper questionnaire was used to collect both qualitative and quantitative data.

Data collection took place at a tertiary hospital in Oman, located in a well-populated area of Muscat. The study setting was the main tertiary teaching hospital that receives patients throughout the country, who require subspecialty evaluation and treatment. This tertiary teaching hospital is the main health care setting in Oman that recruited CNSs in their services since 1998.

### Sample

A convenience sample consisting of nurses working as CNSs in the hospital for a minimum of 1 year and using a smartphone was considered a provision for participation in the study. The study sample size was calculated for the population of CNSs at the tertiary hospital, using the Raosoft web-based sample size calculator (Raosoft Inc) with a confidence level of 95% and a 5% margin of error, and determined to be 42. After obtaining ethical approval, the PI explained the purpose and scope of this study to the nursing administration of the hospital to obtain permission. Once approved, the principal investigator (PI) explained the inclusion criteria and sampling technique to the CNSs at the hospital. The PI distributed the paper questionnaires to participants who met the inclusion criteria. Participation was voluntary. Participants answered individually during work hours and returned the questionnaire to the PI.

### Data Collection

A self-administered questionnaire was adapted from the literature, which evaluated HCPs’ SM use. The tool underwent both internal and external validation before the data collection process. Two expert researchers were consulted to validate and assess the reliability of the content of the tool. The questionnaire was piloted on 5 CNSs to determine the level of understanding, ambiguity or repetition, any difficulties with language or phrasing, comprehension, and length of time to complete the survey. No changes were needed.

The tool consisted of 5 sections. The first section included an outline of the demographic data, CNSs’ professional experience, and the use of smartphones in the work area, using closed-ended questions to assess the frequency of use. The second section investigated the usage of phones and SM using a Likert scale to assess the frequency of use. The third section investigated further the CNS motives for health-related SM use with a focus on the use of the WhatsApp mobile app, as it is the most common SM platform. The fourth section examined the types of barriers (factors that prevent the use of SM) and obstacles (factors that can be overcome or improved by the use of SM) faced by HCPs regarding health-related SM use among CNSs, using a Likert scale. The fifth section was reserved for qualitative assessment with open-ended questions with free-text, extended responses to encourage suggestions for improvements in the use of SM for health-related reasons in the future from the perspective of the participants. To minimize the frequency of missing data, the research team provided participants with clear explanations about completing the questionnaire and answered any of their questions [30].

### Data Analysis

Correlation frequency, percentage, and mean values of the data were analyzed using SPSS software (version 21.0; IBM Corp). Descriptive statistics were used to present the participants’ demographic data, and a distribution was used to represent the data. *P* values were used to test any correlations between 2 variables; that included evaluating years of experience and the use of SM by gender. A *P* level of ≤.05 was considered significant. The associations between some selected independent variables and the use of SM was assessed using a chi-square test. In the Likert scale shown below, the frequency of each response was assessed using a 5-point scale ranging from “Never” to “Always.” The responses were then coded as follows: 1=“Never,” 2=“Rarely,” 3=“Sometimes,” 4=“Often,” and 5=“Always.” On assessing the professional barriers to health-related SM use among CNSs, the Likert scale was coded as follows: 1=“Strongly Disagree,” 2=“Disagree,” 3=“Neutral,” 4=“Agree,” and 5=“Strongly Agree.” Then, the frequency of each response was reported as the percentage of each option. After using the previous coding scheme, the data were analyzed quantitatively. Thematic analysis was performed to analyze participants’ suggestions, which comprised 6 steps: familiarization with the data, generation of initial codes, searching for themes, reviewing of the themes, defining the themes, and finally write-up.

### Ethical Considerations

Ethical approval was attained from the medical research ethics committee at the College of Medicine and Health Science, Sultan Qaboos University (REF.NO.SQU-EC/169/2020, MREC#2205). The data were collected anonymously, and consent was obtained from all participants.

## Results

### Quantitative Findings

Table 1 shows that the surveys were distributed to 47 CNSs, which is the total number of CNSs working in SQUH. Of them, 43 (91.5%) CNSs responded to the questionnaire and 4 (8.5%) did not respond. Thirty-six (83.7%) CNSs were female, and the majority of respondents (n=30, 69.8%) were 30-35 years old. Thirty-nine (90.7%) respondents had more than 10 years of experience. In total, 27 (62.8%) CNSs used 1 smartphone, while the remaining 16 (37.2%) used 2-3 smartphones. All respondents were using SM, of whom 22 (51.5%) were using 4-6 SM apps. There was almost an equal number of participants who spend 1-2 hours (n=18, 41.9%) and those who spend 3-4 hours (n=15, 34.9%) on SM apps to respond to the patients and other HCPs, with 27 (62.8%) CNSs using SM it during work, at home, and at leave times. Only 5 participants reported using hospital Wi-Fi during work time, indicating that the vast majority of the respondents were covering their own phone bills in order to communicate with patients and other HCPs.

**Table 1:** Demographic data of the participants (N=43).

Characteristics		Participants, n (%)
**Gender**		
	Male	7 (16.3)
	Female	36 (83.7)
**Age (years)**		
	30-35	30 (69.8)
	35-40	13 (30.2)
**Years of experience (years)**		
	5-10	4 (9.3)
	>10	39 (90.7)
**Certification**		
	MSc	9 (20.9)
	BSc with a high diploma	5 (11.6)
	BSc	29 (67.4)
**Number of smartphones used^a^**		
	1	27 (62.8)
	2	15 (34.9)
	3	1 (2.3)
**Number of social media apps currently in use**		
	<3	15 (34.9)
	4-6	22 (51.2)
	>6	6 (14)
**Average cost per month for smartphones used for communication with patients and health care providers communication (Omani Rial; 1 Omani Rial=US $2.6)**		
	0	5 (11.6)
	2-10	19 (55.9)
	11-20	14 (32.6)
	21-40	5 (11.6)
**Average hours per day of social media use in responding to patients and health care professionals^a^**		
	1-2	18 (41.9)
	3-4	15 (34.9)
	5-6	9 (20.9)
	>7	1 (2.3)
**Using smartphones for hospital issues**		
	Only during working hours	11 (25.6)
	During work hours and at home	5 (11.6)
	During work hours, at home, and during leave time	27 (62.8)
**Do you prefer health-related social media articles?**		
	Never	1 (2.3)
	Rarely	0 (0)
	Sometimes	21 (48.8)
	Often	18 (41.9)
	Always	3 (7)
**How much do you believe in news on social media?**		
	100%	0 (0)
	80%-90%	11 (25.6)
	50%-70%	15 (34.9)
	<50%	17 (39.5)
**How do you feel when you cannot use social media?**		
	Relieved	18 (41.9)
	Unable to leave the phone	3 (7)
	Afraid that you will miss important news	22 (51.2)
**Have you ever experienced any physical discomfort as a result of using social media?^b^**		
	Yes	34 (79.1)
	No	9 (20.9)
**Have you ever experienced any psychological discomfort as a result of using social media?^b^**		
	Yes	21 (48.8)
	No	22 (51.2)
**Have you ever been humiliated or criticized by social media?**		
	Yes	8 (18.6)
	No	35 (81.4)
**Have you ever sued (by law) any patient, doctor, or any health care professional due to social media humiliation or assaults?**		
	Yes	0 (0)
	No	43 (100)

^a^No significant difference in the number of mobile phones used and the number of hours spent on using social media (*P*=.46).

^b^Significant correlation in the number of clinical nurse specialists who reported physical and psychological discomfort and the number of hours spent in using social media apps (*P*=.003).

Multimedia Appendix 1 shows that the most frequently used SM apps among CNSs in this study were WhatsApp (n=41, 95.3%), Instagram (n=24, 55.8%), and YouTube (n=12, 27.9%), and in comparison, the least used apps were Snapchat (n=4, 9.3%), Twitter (n=9, 20.9%), LinkedIn (n=4, 9.3%), and Facebook (n=4, 9.3%). Yet, a few CNSs in this study use other SM apps (n=1, 2.3%) as well, such as Zoom.

### CNSs’ Main Motives for Using SM

Table 2 displays the 11 motives listed for CNSs to acknowledge the main reasons for using SM. More than half of the CNSs in this study were using SM to increase their knowledge and to communicate with patients and other HCPs (n=37, 86%). In addition, the same number of CNSs also agreed that using SM helped them to reach patients easily (n=32, 74.4%), reduce their number of hospital visits (n=32, 75.0%), increase efficacy (n=33, 76.7%), and increase patient satisfaction (n=34, 79.1%), while around one-third of CNSs were not interested in using SM for publicizing their achievements (n=13, 30.3%).

**Table 2:** Clinical nurse specialists’ motives or reasons for using social media.

Reasons for using social media	Never, n (%)	Rarely, n (%)	Sometimes, n (%)	Often, n (%)	Always, n (%)
Increase knowledge	2 (4.7)	1 (2.3)	9 (20.9)	21 (48.8)	10 (23.3)
Nurse-to-patient communication	0 (0)	4 (9.3)	10 (23.3)	13 (30.2)	16 (37.2)
Nurse-to-doctor communication	0 (0)	2 (4.7)	6 (14)	14 (32.6)	21 (48.8)
Nurse-to-nurse communication	0 (0)	0 (0)	5 (11.6)	18 (41.9)	20 (46.5)
Reaching patients easily	1 (2.3)	3 (7)	7 (16.3)	11 (25.6)	21 (48.8)
Efficacy	0 (0)	4 (9.3)	6 (14)	24 (55.8)	9 (20.9)
Maintaining a good image of the health care institution	2 (4.7)	6 (14)	11 (25.6)	18 (41.9)	6 (14)
Publicizing your specialty achievements or your efficiency (or both)	4 (9.3)	14 (32.6)	12 (27.9)	11 (25.6)	2 (4.7)
Communicating well with other health care providers	0 (0)	0 (0)	6 (14)	20 (46.5)	17 (39.5)
Helping patients reduce the number of hospital visits	4 (9.3)	0 (0)	7 (16.3)	13 (30.2)	19 (44.2)
Increasing patient satisfaction	2 (4.7)	0 (0)	7 (16.3)	17 (39.5)	17 (39.5)

On the other hand, Table 3 shows that the motives for using WhatsApp were extending their network (n=28, 65.1%), updating colleagues about workflow, and sharing information about work with colleagues (n=29, 67.5%). In addition, they were using it to share information on medical conferences, workshops, symposia (n=29, 67.5%), etc. Furthermore, introducing the achievements of their hospital to the outside world was not an area of interest for using SM among 18.6% (n=8) of those using WhatsApp. There was no significant difference in the number of mobile devices used and the number of hours spent using SM (*P*=.46). Moreover, there was no significant difference between the educational level (in terms of certification) and the number of hours spent using SM (*P*=.57).

**Table 3:** Clinical nurse specialists’ motives for health-related social media use at the item level for WhatsApp.

Clinical nurse specialists’ motives for health-related social media use at the item level for WhatsApp	Never, n (%)	Rarely, n (%)	Sometimes, n (%)	Often, n (%)	Always, n (%)
Extending the network with colleagues	1 (2.3)	2 (4.7)	12 (27.9)	15 (34.9)	13 (30.2)
Updating colleagues about workflow	1 (2.3)	3 (7)	10 (23.3)	15 (34.9)	14 (32.6)
Presenting the hospital to the outside world	1 (2.3)	7 (16.3)	17 (39.5)	12 (27.9)	6 (14)
Sharing information on medical conferences, workshops, and symposia with others	1 (2.3)	1 (2.3)	12 (27.9)	15 (34.9)	14 (32.6)

### Professional Barriers and Obstacles Regarding Health-Related SM Use

Table 4 shows that time constraints (n=26, 60.4%), privacy concerns (n=26, 60.5%), and unawareness about the legal grounds (n=22, 51.2%) of SM use were the main barriers faced by more than half of the CNS. In addition, there were obstacles faced by two-third of the CNSs in using SM, such as receiving messages from patients whom they did not know who were asking for help (n=29, 67.4%), difficulties in receiving videos, images, or electrocardiographs (n=28, 65.2%), etc, from patients via SM, and lack of landlines (n=28, 65.2%).

**Table 4:** Professional barriers regarding health-related social media use among clinical nurse specialists.

Professional barriers regarding health-related social media use	Strongly disagree, n (%)	Disagree, n (%)	Neutral, n (%)	Agree, n (%)	Strongly agree, n (%)
Inefficiency (uncertainty about the trustworthiness of social media applications and gives patients the wrong idea when they see a clinical nurse specialist holding a phone)	0 (0)	6 (14)	17 (39.5)	14 (32.6)	6 (14)
Lack of skills	5 (11.6)	12 (27.9)	16 (37.2)	10 (23.3)	0 (0)
Legal ground (not knowing the legal aspects of social media)	1 (2.3)	6 (14)	14 (32.6)	18 (41.9)	4 (9.3)
Privacy concern	0 (0)	4 (9.3)	13 (30.2)	20 (46.5)	6 (14)
No need for its use	8 (18.6)	21 (48.8)	8 (18.6)	6 (14)	0 (0)
Time constraints	0 (0)	9 (20.9)	8 (18.6)	25 (58.1)	1 (2.3)
Receiving messages from patients whom you do not know, or from patients seeking help from practitioners of other medical specialties	0 (0)	4 (9.3)	10 (23.3)	21 (48.8)	8 (18.6)
Need to receive videos from patients	1 (2.3)	3 (7)	11 (25.2)	22 (51.2)	6 (14)
Lack of landlines or busy landline	0 (0)	2 (4.7)	11 (25.2)	2 (51.2)	6 (14)

Furthermore, as shown in Table 1, overall, 79.1% of CNSs listed that they were experiencing some physical discomforts such as insomnia, carpel tunnel syndrome, eye dryness, eye pain, headache, joint pain, wrist pain, nausea, neck pain, and thumb pain as a result of using SM applications. Furthermore, 21 (48.8%) CNSs reported experiencing some psychological discomfort including anxiety, sadness, stress, and anger. There was noticeable significance in the number of CNSs who reported physical and psychological discomfort and the number of hours spent in using SM applications (*P*=.003). Nonetheless, 22 (51.2%) CNSs reported that they felt afraid of missing something if they did not use SM. Furthermore, less than a quarter of the study participants reported that they were humiliated by SM either by a patient or an HCP (n=8, 18.6%) but no one has ever sued a patient or HCPs due to SM humiliation or assault.

### Qualitative Findings

The open-ended questions asked about participants’ suggestions for the future use of SM among CNSs at SQUH. Indeed, the CNSs proposed new ideas, which they thought (if applied) would help regulate and develop the use of SM in the health care setting. Two key themes from these data emerged: organizational support and mitigating the risks of SM.

### Organizational Support

The CNSs in this study were aware of the benefit of the SM in their daily work; therefore, they suggested, “the organisation could add the WhatsApp platform in the hospital computers for easy access and conversation tracking.”

In addition, half of the CNS suggested that the “organisation could have a formal account in SM, that all the employee could use for health education, hospital orientation, hospital workflow instruction, and guidance for the community and give a message to users that this is being monitored.”

Half of participants suggested having “an official specialized platform to interact with their patients and conduct virtual clinics.”

Other participants suggested, “the organisation provide a mobile phone to CNSs with high patients’ number to facilitate direct communication when it’s needed. Providing free, strong and fast WIFI network coverage was suggested by the participants to encourage CNSs to use SM.”

### Mitigating Risks of SM

The CNSs’ concerns were compounded by their experiences and lack of knowledge about the legal aspect of using SM. Therefore, they suggested, “a focal person (who knows the legal aspects) in the organisation to refer to when they are faced with a legal issue related to SM.” Other CNSs suggested, “clear regulation and guidance in the use of SM including legal and professional instructions.” Some believed that health care institutions should regulate the usage of SM activities and “the Hospital Information System to programme the most used SM platforms (like WhatsApp) for an automatic phone response to patients who are calling the CNS during their leave or when they are off duty and redirecting them to the HCP who is covering that CNS.”

## Discussion

### Principal Findings

This was the first study reporting the use of SM applications by CNSs in Oman and the Middle East in order to acknowledge the motives, barriers, and future aspects of SM usage. The results of this study showed that all participants used SM in the hospital, and WhatsApp was the most frequently used application. SM was mostly used to communicate with patients or to increase their knowledge. However, there were 2 main concerns regarding the use of SM: privacy and legal concerns.

The results of this study show that all respondents were engaged with SM and were using 1 or more SM apps on their phones, which is similar to the findings of Wang et al [13]. However, Surani et al [31] reported that 87.9% of the nurses in their study were SM users; that can be explained by the time difference between the 2 studies [31].

Our results show that CNSs were spending 1-2 hours per day on SM use. These findings are similar to those of a study undertaken by the University of North Texas [31]. While the average daily use for SM among Omanis is 6 hours per day according to a previous study conducted by Al-Kindi [22], this might be explained by the difference in the age group and the purpose of using the SM platform.

Our results also show that WhatsApp is most preferred SM application used in the clinical area because it is free, easy to use, and has an international pool of users. Indeed, a study from the Kingdom of Saudi Arabia revealed that Twitter, YouTube, Instagram, Facebook, Snapchat, and LinkedIn were the most frequently used SM platforms, in descending order, for professional development among HCPs [32]. This indicates that SM has the potential to be a multifocal platform through which every segment of the society can have their voices heard, facilitating knowledge exchange and improving networking opportunities [33].

The top benefits that CNSs agreed on for using SM apps on smartphones were that they offered opportunities that cannot be achieved through the use of regular landlines, and other benefits have been anecdotally reported in the wider literature [14,15,34]. Moreover, it was reported that SM is a tool for the health care community to improve quality and professional practice at a low cost [35]. When communicating with user patients, SM allows for real-time interaction and provides tools and spaces to access and exchange information and to improve opportunities for participation and expression. The health professional networks are used to disseminate results, discussions, and networking for outreach and research [14,15,34]. According to Wang et al [13] SM will soon be the preferred communication tool for health care corporations. In regard to hospital organizations, the available studies were not conclusive concerning the use of SM. However, there are hospitals with a highly visible profile and with brand recognition on SM platforms, such as the Mayo Clinic, the Cleveland Clinic, and Mount Sinai Hospital, all of which are in the United States [36]. Overall, despite the widespread commitment to SM, hospitals continue to undercommunicate their corporate identity, with little variety in the range of SM used. This is in line with the poor use of content and applications in the context of limited professionalization of the corporate presence on SM [37].

Regardless the benefits of SM use, CNSs were always pressured with time restrictions; this could be because nurses are always busy in the clinical area with administrative and clinical jobs. The second barrier was confidentiality. Patients’ private information can be interspersed and might reach other family members of the patient [38]. The risk borne by commenting on the internet about any patient condition or having excessive trust in SM when connecting with HCPs regarding patient health status, and the neglect of other traditional communication channels are also seen as barriers. Given this situation, Koehler et al [38] suggested that providing staff with smartphones from the institution could address these restrictions. The third barrier was unawareness about the legal grounds of SM use in clinical practice [38]. This problem was also seen in most of the hospitals worldwide [31]. For example, Surani [31] reported that doctors were more aware of legal policy for SM use in their hospitals as compared to nurses. This is an area that needs to be highlighted to the CNSs by the hospital administration, using clear guidelines that need to be established at the institution. The fourth barrier was inefficiency [31]. CNSs were concerned when the clients saw them holding their phone, they might perceive them to be using the phone for a nonwork purpose. In fact, Koehler et al [38] explained this perception in a way that HCPs may think that patients are not aware that smartphones can be used for medical purposes. Most of the CNSs mentioned that some people felt that there was no need to use SM in clinical practice [38]. However, 2 key reasons that SM use was seen as beneficial were (1) landlines were difficult to locate, which make it time-consuming when searching for a free landline to communicate with patients; and (2) the necessity of some specialties to receive some images, electrocardiographs, videos, laboratory results, x-ray images, reports, etc, from their patients to help them in the continuity of care and reduce the number of hospital visits.

Most of the CNSs preferred health-related SM articles but one-third of them did not trust whatever they read. Prasad [39] described that being cautious on the topic of the health information on the internet originated from unregulated sources and is an important consideration. Because the source is not always well defined or is not stated and the messages are not clear or are not well referenced, people question the reliability of the content disseminated on SM [40].

A high percentage of physical symptoms was identified among CNSs as a result from extensive use of SM. Velthoven et al [41] described the reason as increased use of the thumb to text and operate smartphones can damage the thumb muscle, causing tenosynovitis. This condition led to the coining of a recent medical term called “Whatsappitis.” Furthermore, “text neck” is another term generated to define tilting the head forward for prolonged times, thus forcing the neck muscles, ligaments, and tendons to strain when using smartphones along with SM texting. In addition to that, the prolonged use of SM platforms can lead to weakness in the hand muscles, tenosynovitis, nerve compression, and chronic neck pain [41].

Nearly half of the CNSs reported having psychological symptoms as a result of using SM. Studies have shown that compulsive SM use significantly triggered fatigue, which later results in elevated anxiety and depression [42]. Likewise, Dhir et al [43] reported the elevation of emotional fatigue, which later translates to poor physical performances and perceptions among Taiwanese Nurses.

Despite SM-induced physical and psychological discomfort, more than half of the CNSs reported that they continued using it. That condition was defined as fear of missing out (FoMO)—it is an apprehension or concern of being disconnected, absent from, or missing an experience which others (ie, peers, friends, and family) might receive or enjoy [44]. Our findings suggest that FoMO may be stemming from the responsibility of the CNSs. They do not want to miss information about patient health progress or administrative updates. Nevertheless, FoMO was found to be associated with negative health outcomes [45].

Although some of the CNSs were humiliated through SM, this study outcome indicated that none of them have taken legal action toward the aggressor. An elucidation of that by the limited knowledge of the HCPs about the legal aspects of SM use is also apparent in the wider literature [31]. In fact, a law on cybersecurity in Oman‎‎ was issued in accordance with Royal Decree number 12/2011 of the Information Technology Crimes Act in Oman. The decree covers legal action to be taken in the following ‎cases:‎ infringement of the integrity, confidentiality, and availability of electronic data and information and information systems in all its forms, misuse of IT, forgery and information fraud, and content crimes [46]. We recommend teaching the HCPs the legal aspects and consequences.

### Strengths and Limitations

The main strength of this study is the use of a mixed methods design, which allowed achieving the aim of the study. Moreover, it is novel to the Omani context. The limitations of the study are its small sample size, which was due to the small number of CNSs working at SQUH, which limits the generalization of results to the larger population. The application of the conclusions of the study may be limited by time and geographical location, which further limits its generalizability.

### Recommendations

Future research should consider a larger sample size for similar studies to enhance the generalizability of our results. Moreover, examining SM usage and perception among patients and general nurses in other settings is recommended. Finally, health policy makers should consider addressing the legal aspects of SM usage in health care settings.

### Conclusions

SM proved to be a powerful institutional communication and health educational tool. However, this is accompanied by risks to patients’ privacy, time constraints, and efficacy. Critically, the health institution has to regulate the use of SM among HCPs and teach them how to minimize potential risks considering the legal aspects of SM usage in health care settings. The legal aspect and consequences should be documented in hospital policy, and a focal person should be allocated a role to help the staff who need support in that field.

## Appendix

Multimedia Appendix 1 The most frequently used social media apps among clinical nurse specialists.

## Abbreviations

APN: advanced practitioner nurse
CNS: clinical nurse specialist
FoMO: fear of missing out
HCP: health care professional
PI: principal investigator
SM: social media
SQUH: Sultan Qaboos University Hospital

## Data Availability

The data sets generated during and analyzed during this study are available from the corresponding author on reasonable request.
