# Supplementary figures and images for: The Use of Social Media by Clinical Nurse Specialists at a Tertiary Hospital: Mixed Methods Study
Source: JMIR Nurs. 2023 Aug 24;6:e45150. doi: 10.2196/45150 (PMC10485714; doi:10.2196/45150)

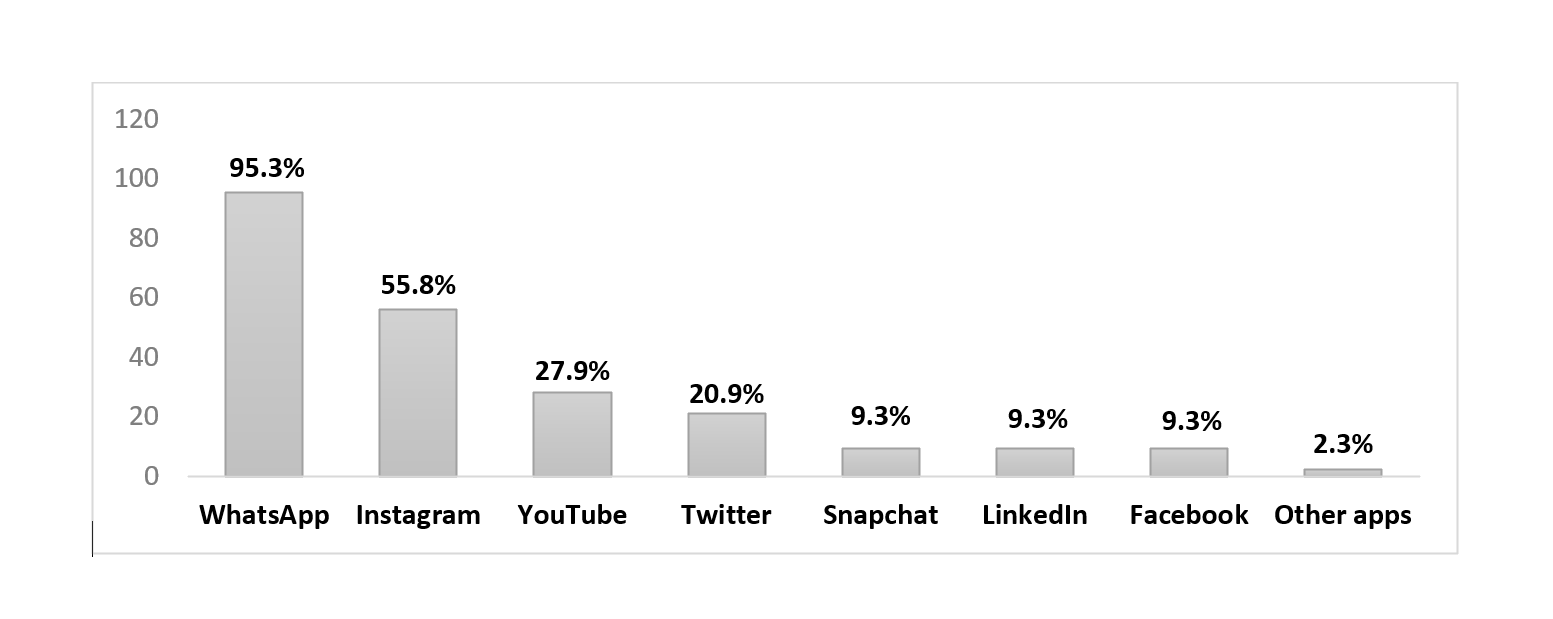

Supplement: Multimedia Appendix 1 [file nursing_v6i1e45150_app1.png]
